# Efficacy and safety of thymosin combined with anticancer therapy for colorectal cancer: a systematic review and meta-analysis of controlled studies

**DOI:** 10.3389/fonc.2026.1957041

**Published:** 2026-10-29

**Authors:** Guannan Zhang, Yuxin Zhou, Tao Sun, Xiaoqi Chen, Yuling Zheng

**Affiliations:** 1 Department of Oncology, the First Affiliated Hospital of Henan University of Chinese Medicine, Zhengzhou, China; 2 Henan University of Chinese Medicine, Zhengzhou, China; 3 Department of Cardiovascular Disease, The First Affiliated Hospital of Henan University of Chinese Medicine, Zhengzhou, China

**Keywords:** colorectal cancer, meta-analysis, thymalfasin, thymopentin, thymosin alpha 1, trial sequential analysis

## Abstract

**Objective:**

To evaluate the efficacy and safety of thymosin preparations added to the same background anticancer treatment in patients with colorectal cancer.

**Methods:**

We searched nine bibliographic and trial-registration sources through 1 October 2026. Randomized controlled trials (RCTs) and nonrandomized studies with concurrent controls were eligible. Risk of bias was assessed using RoB 2 and ROBINS-I. RCTs provided the primary evidence; nonrandomized studies were analyzed separately. Review Manager 5.4.1 calculated risk ratios (RRs) or mean differences (MDs) with 95% confidence intervals (CIs), organized by treatment setting. Sensitivity analyses, GRADE and exploratory trial sequential analysis (TSA) assessed robustness and uncertainty.

**Results:**

Thirty studies involving 2,514 participants comprised 25 RCTs (2,079 participants) and five nonrandomized studies (435 participants). In postoperative RCTs, CD4 percentage (MD 6.33, 95% CI 4.89 to 7.76), CD4/CD8 ratio (MD 0.25, 0.16 to 0.35) and NK-cell percentage (MD 6.41, 3.72 to 9.11) were higher with thymosin; CD3 and CD8 findings were inconclusive. KPS was higher in two postoperative RCTs (MD 8.50, 6.52 to 10.48) and two advanced or mixed-stage trials (MD 4.35, 1.90 to 6.80), with source-reporting concerns affecting the latter. Postoperative neutropenia was inconclusive in the RCT (RR 0.91, 0.82 to 1.01), whereas two nonrandomized studies reported a lower unadjusted risk (RR 0.65, 0.51 to 0.83). Comparable one-year survival evidence came from one trial. Certainty was low or very low.

**Conclusion:**

This review identifies RCT-based signals in circulating immune indices and performance status and clarifies the contribution of nonrandomized evidence. These findings inform the selection of outcomes and treatment settings for future trials. Current evidence is insufficient to support routine clinical use of thymosin preparations in colorectal cancer.

**Systematic review registration:**

https://www.crd.york.ac.uk/PROSPERO/view/CRD420261467566, CRD420261467566.

## 1 Introduction

Colorectal cancer is a leading cause of cancer incidence and mortality worldwide (1). Management of localized colon cancer is guided by pathological stage and resectability (2, 3). Rectal and metastatic disease management additionally considers tumor site, molecular profile, and treatment intent (4, 5). Surgery remains the foundation for localized disease, while oxaliplatin-fluoropyrimidine combinations are central to adjuvant treatment (6, 7). Total neoadjuvant approaches are increasingly used for locally advanced rectal cancer (8). Biomarker-directed targeted and immune therapies have also transformed selected metastatic and mismatch repair-deficient settings (9, 10).

This precision framework also clarifies why host immune status may matter. Colorectal cancer comprises biologically distinct molecular and immune phenotypes (11). The density and spatial distribution of tumor-infiltrating T cells provide prognostic information beyond conventional staging (12, 13). Baseline circulating lymphocyte subsets track clinically relevant heterogeneity (14, 15). Primary tumor sidedness likewise carries clinically relevant prognostic and predictive information (16). However, circulating percentages cannot be assumed to represent intratumoral immunity.

Thymosin preparations include thymosin alpha 1 (thymalfasin), thymopentin, and heterogeneous thymic peptide injections (17–19). Experimental evidence indicates that thymosin alpha 1 can promote dendritic-cell maturation and influence Toll-like receptor and MyD88-dependent signaling (20, 21). It can also support T-cell responses (22). It has additionally been shown to modulate macrophage polarization (23). These findings provide a rationale for evaluating thymosin as an adjunct to anticancer treatment, particularly when surgery, chemotherapy, or radiotherapy perturbs immune reserve.

A broad Cochrane review of thymic peptides in cancer found limited evidence for definitive survival or tumor-response benefits and highlighted clinical and methodological heterogeneity (24). Colorectal-cancer-specific controlled studies have since accumulated across perioperative care, chemotherapy and recovery after treatment. We therefore systematically evaluated thymosin preparations added to matched anticancer treatment. By synthesizing randomized and nonrandomized evidence separately and integrating sensitivity analysis, GRADE and TSA, this review aims to distinguish potential treatment signals from uncertainties that should guide further clinical investigation.

## 2 Methods

### 2.1 Reporting framework and protocol status

This systematic review was reported according to PRISMA 2020 and conducted with reference to the Cochrane Handbook (25, 26). The protocol was registered in PROSPERO (CRD420261467566). White-cell-count synthesis and TSA of liver dysfunction and thrombocytopenia were exploratory analyses.

### 2.2 Eligibility criteria

Eligible participants had pathologically diagnosed colon or rectal cancer at any stage. Interventions were injectable thymosin alpha 1/thymalfasin, thymopentin, or thymic peptide preparations added to surgery, chemotherapy, radiotherapy, targeted therapy, or immunotherapy, including recovery or maintenance after matched anticancer treatment. Controls received the same background treatment without thymosin. RCTs and nonrandomized studies with concurrent controls were eligible. Exclusions comprised single-arm studies, healthy-person comparators, duplicate reports, materially unequal background treatment, inseparable mixed-cancer populations, and reports without usable between-group outcome data. Oral sequential regimens and maintenance based solely on traditional herbal treatment were outside the intervention scope.

### 2.3 Information sources and search strategy

PubMed, Embase, the Cochrane Library, Web of Science Core Collection, CNKI, Wanfang, VIP, SinoMed, and ClinicalTrials.gov were searched for reports published or registered through 1 October 2026. No language, study-design, outcome, or full-text-availability filters were applied. Controlled vocabulary and free-text terms for colorectal neoplasms were combined with thymosin, thymalfasin, thymopentin, thymic peptides or extracts, and relevant trade names. Reference lists and related reports were also examined. Bibliographic and trial-registration records were combined for screening.

### 2.4 Study selection and report families

All records were combined and deduplicated before screening titles, abstracts, and keywords. Two reviewers independently assessed potentially eligible full texts, resolving disagreements through discussion or third-reviewer adjudication. Reports from the same cohort were linked and counted as one study, with the most complete report used for each outcome. Full-text exclusions were classified according to the eligibility criteria.

### 2.5 Data extraction and outcomes

Study design, sample size, sex, recruitment center and period, tumor site and stage, resection or metastatic status, treatment intent, thymosin formulation and dose, background treatment, duration, follow-up and assessment times were extracted. Adjustment variables and adjusted effects were recorded for nonrandomized studies when available. Binary data comprised events and group totals; continuous data comprised means, standard deviations and sample sizes. Source pages and table or figure locations were recorded. Percentages and absolute counts, post-treatment values and changes, and different toxicity grades were distinguished. Each study contributed one eligible comparison and one assessment per synthesis. Outcomes with unresolved denominators, dispersion measures or scale definitions were summarized narratively. NR denotes relevant information not reported; NA denotes information not applicable.

### 2.6 Risk-of-bias assessment

Two reviewers independently applied RoB 2 to the RCTs, assessing the effect of assignment to intervention across five domains: randomization, deviations from intended interventions, missing outcome data, outcome measurement, and selection of reported results (26, 27). Assessments concerned a specified outcome and time point, prioritizing survival or recurrence when reported, followed by clinical outcomes and immune markers. Nonrandomized studies were assessed using ROBINS-I 2016 (28). Relevant confounders included disease stage, age, performance status, baseline immune function, tumor site, treatment regimen and cycles, comorbidity, nutrition, and other immunomodulatory treatment. Disagreements were resolved by consensus or adjudication.

### 2.7 Statistical analysis, subgrouping, and sensitivity analysis

Review Manager 5.4.1 calculated effect estimates (29). RCTs formed the primary analyses, and nonrandomized studies were synthesized separately; no cross-design summary effect was calculated. Binary outcomes used RRs with Mantel–Haenszel methods and continuous outcomes used MDs with inverse-variance methods, each with 95% CIs. I² quantified heterogeneity (30). RCT analyses used a fixed-effect model when I² was ≤50% and a DerSimonian–Laird random-effects model otherwise. Nonrandomized syntheses used random-effects models. Adjusted estimates were preferred when available; all nonrandomized comparisons used here were unadjusted and were interpreted as associations. Postoperative treatment was analyzed separately from advanced, metastatic or mixed-stage disease. The latter category included reports from which stage-specific results could not be separated. Analyses summarized the broader adjunctive-treatment question, with formulation, regimen and assessment differences considered in interpretation. At least two studies with usable outcome data were required for pooling. Forest plots were generated in RevMan, with R 4.6.1 used for figure assembly and risk-of-bias plots. Perioperative surgery without concurrent chemotherapy, radiotherapy-specific assessments, and prolonged maintenance or recovery after completed treatment were described separately when their outcomes could not represent the chemotherapy-period comparisons.

Leave-one-out analyses were performed for outcomes with at least three studies, using StataNow/MP 19.5 for RCTs and RevMan for nonrandomized studies, retaining the primary model after each omission (31). Random-effects RRs in Stata used inverse-variance DerSimonian–Laird estimation. Additional RevMan analyses excluded studies with unclear assessment timing and examined the influence of source-reporting concerns. For KPS, Song 2013 and Zhou 2015 were omitted separately and together. For white-cell counts, the early assessment-window analysis excluded the trial averaging measurements over two cycles. Single-study results were not described as pooled estimates. The number of studies within clinically coherent strata was insufficient for reliable formulation comparisons or meta-regression.

### 2.8 Certainty of evidence

GRADE assessed certainty separately for RCT and nonrandomized evidence across risk of bias, inconsistency, indirectness, imprecision and publication bias (32). RCT evidence started at high certainty and nonrandomized evidence at low certainty. Downgrading considered incomplete allocation and analysis reporting, source-reporting concerns, residual confounding, unexplained heterogeneity, sparse information and CIs compatible with materially different effects. Certainty concerned the measured outcome; circulating immune indices were not regarded as validated surrogates for clinical benefit.

### 2.9 Trial sequential analysis

TSA 0.9.5.10 Beta used a two-sided type I error of 5%, 80% power, chronological trial accumulation and O’Brien–Fleming monitoring boundaries (33). Only RCT outcomes with at least three contributing studies were eligible. Exploratory analyses of liver dysfunction and thrombocytopenia in advanced or mixed-stage disease used a 20% relative risk reduction and the pooled control-event proportion, with observed-effect sensitivity analyses. Continuous-outcome TSAs used empirical effect sizes and pooled within-group variances. Required information size (RIS), accrued information and boundary crossing were evaluated. No TSA was performed for survival, KPS or postoperative neutropenia because fewer than three RCTs contributed to each. TSA was interpreted alongside clinical heterogeneity, risk of bias and GRADE certainty.

### 2.10 Publication bias and meta-regression

Funnel plots, Egger regression, trim-and-fill, and meta-regression were planned only for outcomes with at least ten studies (34). No outcome reached that threshold, so these analyses were not performed.

## 3 Results

### 3.1 Study selection

The searches identified 1,110 records: 176 from PubMed, 244 from Embase, 101 from Web of Science, 53 from CNKI, 405 from Wanfang, 57 from VIP, 49 from SinoMed, 18 from the Cochrane Library, and seven from ClinicalTrials.gov. After removal of 332 duplicates, 778 records underwent title, abstract, and keyword screening, and 698 were excluded. All 80 reports sought for full-text assessment were obtained. Fifty reports were excluded, leaving 30 studies for inclusion (**Figure 1**).

**Figure 1:**
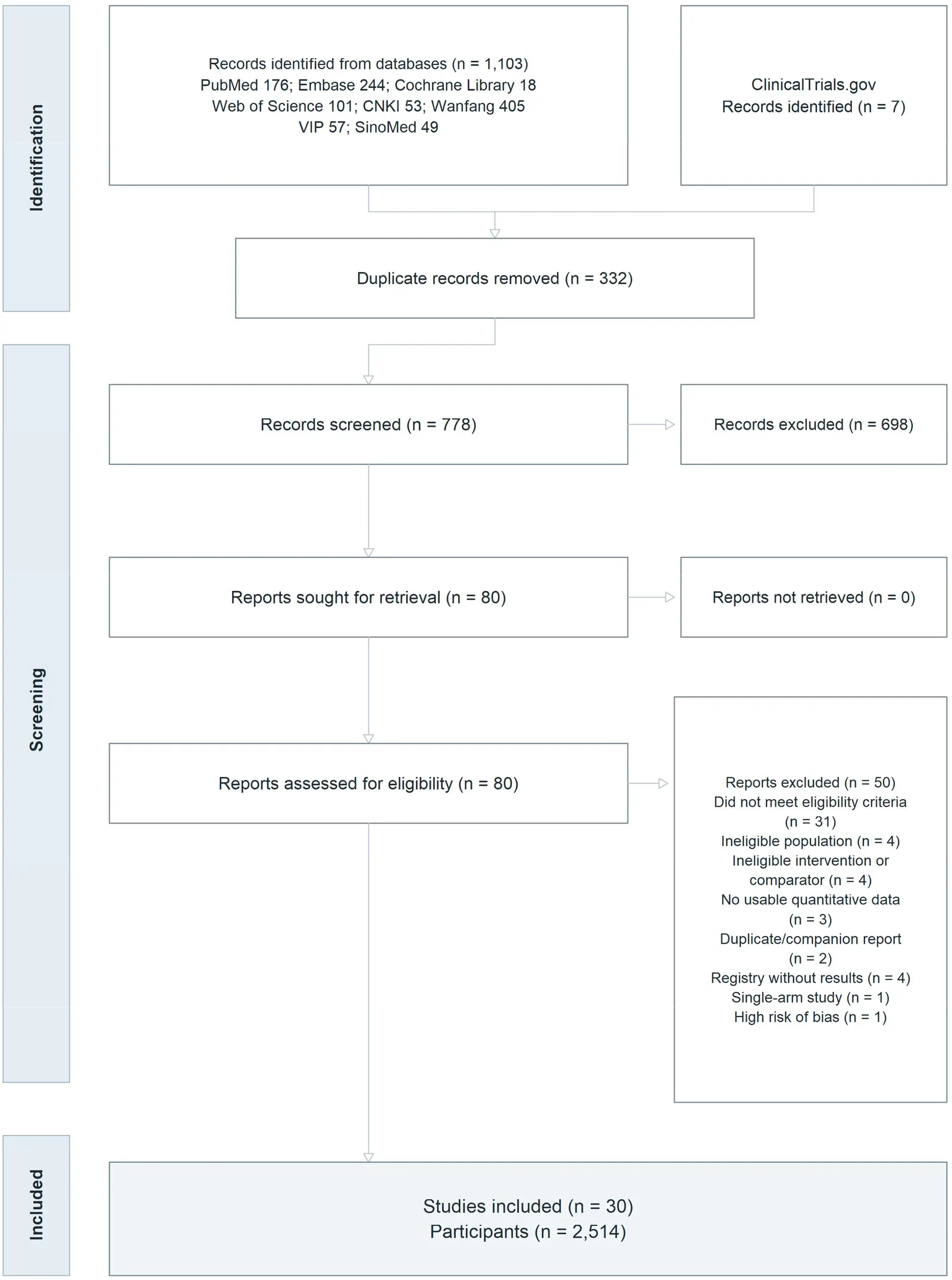
PRISMA 2020 flow diagram of study selection.

### 3.2 Study characteristics and risk of bias

The 30 studies enrolled 2,514 participants in eligible comparisons: 1,282 received thymosin-containing treatment and 1,232 received matched control treatment. Twenty-five RCTs enrolled 2,079 participants (1,058/1,021), and five nonrandomized studies enrolled 435 (224/211). Settings included perioperative care, postoperative chemotherapy, maintenance or recovery after treatment, advanced or metastatic disease, and chemoradiotherapy. Preparations included thymosin alpha 1/thymalfasin, thymopentin and thymic peptide injections. Only the eligible thymosin and control arms of multiarm trials contributed (63, 64). **Tables 1** and **2** report the clinical characteristics, including the available sex distribution, center, recruitment period, disease extent, treatment intent and assessment times. No usable adjusted between-group estimate was identified for the nonrandomized outcome analyses.

**Table 1:** Characteristics of the 30 controlled studies included in the systematic review.

Author and reference	T	C	Mean age T	Mean age C	Stage T	Stage C	Intervention T	Intervention C	Radiotherapy dose	Chemotherapy cycles	Outcomes	Primary endpoint
Ren 2024 (Nonrandomized) (35)	56	50	52.84 ± 6.79	53.26 ± 5.28	II 38; III 18	II 30; III 20	XELOX for 6 cycles plus thymosin alpha 1, 1.6 mg SC every other day	XELOX for 6 cycles	NA	6	⑥⑧⑨⑩⑬	Immune function and safety
Yang 2025 (RCT) (36)	51	51	59.36 ± 4.84	59.87 ± 5.13	High-risk stage II rectal cancer after radical resection	High-risk stage II rectal cancer after radical resection	FOLFOX plus thymosin alpha 1, 1.6 mg SC twice weekly to chemotherapy completion	FOLFOX	45-50.4 Gy in 25–28 fractions before surgery in both groups	6 months	①④⑥⑦⑨⑩⑬	Immune function and recurrence/metastasis
Zhang 2023 (RCT) (37)	120	120	64.91 ± 4.62	65.61 ± 4.90	Stage III or IV after radical resection	Stage III or IV after radical resection	XELOX plus thymosin alpha 1, 1.6 mg SC every other day	XELOX	NA	6-8	①②③⑥⑧⑨⑩⑬	Three-year PFS, OS, and toxicity
Huang 2019 (RCT) (38)	25	25	58.2 ± 7.1	57.5 ± 7.6	Stage II-IIIB rectal cancer after surgery	Stage II-IIIB rectal cancer after surgery	XELOX plus thymopentin, 1 mg/kg SC daily for 3 weeks	XELOX	NA	One observed course	⑧⑨	Blood counts and quality of life
Yang 2018 (RCT) (39)	28	27	NR	NR	II/III overall 25/30; colorectal	II/III overall 25/30; colorectal	FOLFOX4 plus thymosin injection, 200 mg IV on days 1–5 of each cycle	FOLFOX4	NA	12	⑬	Treg proportion and Foxp3/CD127 mRNA
Liu 2018 (RCT) (40)	31	31	35–75	35–75	Stage IV colorectal cancer	Stage IV colorectal cancer	XELOX plus thymosin alpha 1, 1.6 mg SC twice weekly	XELOX	NA	4	⑥⑧⑩⑬	Th17/Treg, KPS, and clinical benefit
Chen 2018 (Nonrandomized) (41)	40	41	NR	NR	Postoperative chemotherapy for colorectal cancer	Postoperative chemotherapy for colorectal cancer	FOLFOX or CAPEOX plus thymalfasin, 1.6 mg SC twice weekly until chemotherapy completion	Matched FOLFOX or CAPEOX	NA	NR	⑥⑦⑧	Immune function and KPS
Xu 2016 (Nonrandomized) (42)	21	21	NR	NR	Perioperative rectal cancer	Perioperative rectal cancer	Standard perioperative care plus thymopentin, 2 mg IM daily for 7 days	Standard perioperative care	NA	NA	⑥⑦⑪	Postoperative immune recovery
Zhou 2015 (RCT) (43)	25	25	51.3 ± 6.3	47.5 ± 5.9	Stage IV colorectal cancer	Stage IV colorectal cancer	XELOX plus thymopentin, 10 mg IM daily for 15 days per course, 4 courses	XELOX	NA	8	⑤⑧⑨⑩	Clinical benefit and toxicity
Liang 2015 (RCT) (44)	20	20	55 (42–68)	54 (40–70)	Stage II-III colon cancer after surgery	Stage II-III colon cancer after surgery	Thymosin alpha 1 maintenance, 1.6 mg SC twice weekly after the same adjuvant chemotherapy	Observation after the same adjuvant chemotherapy	NA	Prior FOLFOX/XELOX; maintenance 3–6 months	①③⑥⑦⑧	Immune function, KPS, and survival
Song 2013 (RCT) (45)	40	40	62.3 ± 7.1	60.0 ± 4.2	Stage I-IV colorectal cancer	Stage I-IV colorectal cancer	XELOX plus thymopentin, 10 mg IM daily for 15 days per course, 4 courses	XELOX	NA	4	③⑥⑦⑧	One-, two-, and three-year survival; immune function
Shi 2013 (RCT) (46)	60	60	30–70 (category distribution)	30–70 (category distribution)	Stage III colon cancer	Stage III colon cancer	FOLFOX4 plus thymosin alpha 1, 1.6 mg SC every other day	FOLFOX4	NA	2	⑥⑦⑧	Immune function and KPS
Chai 2012 (RCT) (47)	19	19	NR	NR	Advanced colorectal cancer	Advanced colorectal cancer	XELOX plus thymopentin, 10 mg daily for 21 days	XELOX	NA	Chemotherapy cycles 3–4 at evaluation	⑥⑦	Immune function
Dai 2012 (RCT) (48)	32	32	Median 51 overall	Median 51 overall	Stage II-IIIB colorectal cancer after surgery	Stage II-IIIB colorectal cancer after surgery	XELOX plus thymosin alpha 1, 1.6 mg SC twice weekly for 3 weeks	XELOX	NA	Short-term observation	⑥⑦⑧⑨	Immune function, KPS, and blood counts
Sheng 2011 (RCT) (49)	38	36	52	49	Mixed resected, unresectable, or recurrent colorectal cancer	Mixed resected, unresectable, or recurrent colorectal cancer	FOLFOX4 plus thymosin alpha 1, 1.6 mg SC three times weekly	FOLFOX4	NA	4	⑥⑦	Immune function
Tao 2009 (RCT) (50)	22	20	Median 66 overall	Median 66 overall	Stage IIA-IIIB colorectal cancer in older adults after surgery	Stage IIA-IIIB colorectal cancer in older adults after surgery	LV/5-FU plus thymosin alpha 1, 1.6 mg SC three times weekly for 3 months per year for 3 years	LV/5-FU	NA	6	④⑥	Recurrence prevention and immune function
Long 2008 (RCT) (51)	28	10	31–68 overall	31–68 overall	Stage III-IV rectal cancer	Stage III-IV rectal cancer	5-FU/MMC/ADM plus thymopentin, 1 mg IM daily	5-FU/MMC/ADM	NA	3	⑤⑥⑦⑨⑩	Tumor response and immune function
Huo 2011 (RCT) (52)	38	38	Median 58 overall	Median 58 overall	Stage IIB-IIIB colorectal cancer after surgery	Stage IIB-IIIB colorectal cancer after surgery	FOLFOX4 plus thymosin alpha 1, 1.6 mg SC daily through 1 week after cycle 2	FOLFOX4	NA	2	⑥⑦⑨	Immune function and leukopenia
Wu 2017 (RCT) (53)	40	40	Overall 46.5 +/- 6.5 (range 40-79)	Overall 46.5 +/- 6.5 (range 40-79)	Advanced colorectal cancer	Advanced colorectal cancer	XELOX plus thymopentin, 1 mg IM twice daily for 14 days	XELOX	NA	2	⑨⑩	Chemotherapy-related toxicity
Yan 2023 (RCT) (54)	39	41	NR	NR	Postoperative chemoradiotherapy for rectal cancer	Postoperative chemoradiotherapy for rectal cancer	IMRT plus capecitabine and thymalfasin, 1.6 mg SC twice weekly	IMRT plus capecitabine	IMRT; dose reported in thesis	Concurrent with radiotherapy	⑥⑦⑧⑨⑫	Acute radiation enteritis
Shi 2018 (RCT) (55)	30	30	NR	NR	Colorectal cancer receiving chemotherapy	Colorectal cancer receiving chemotherapy	XELOX plus thymalfasin, 1.6 mg SC twice weekly	XELOX	NA	2	⑥⑬	Circulating tumor cells and immune function
Yao 2024 (Nonrandomized) (56)	13	13	NR	NR	Locally advanced lower rectal cancer	Locally advanced lower rectal cancer	Neoadjuvant chemoradiotherapy plus tislelizumab and thymalfasin, 1.6 mg twice weekly	Neoadjuvant chemoradiotherapy plus tislelizumab	NR	NR	⑤⑫	Pathological response and safety
Niu 2024 (RCT) (57)	200	200	64.44 ± 17.50 / 64.88 ± 16.26	65.52 ± 17.15	cT1+ or node-positive colorectal cancer	cT1+ or node-positive colorectal cancer	Surgery plus XELOX and thymalfasin, 1.6 mg SC twice or three times weekly for 6 months	Surgery plus XELOX	NA	6	②④⑥⑪	Infection, complications, and three-year prognosis
Sha 2024 (Nonrandomized) (58)	94	86	NR	NR	Postoperative colorectal cancer	Postoperative colorectal cancer	XELOX for 4 cycles plus thymosin alpha 1, 1.6 mg SC three times weekly	XELOX for 4 cycles	NA	4	④⑥⑧⑨⑩⑬	Immune function, tumor markers, and recurrence
Xu 2004 (RCT) (59)	29	20	65.7 ± 13.6	63.2 ± 10.0	Dukes B2/C after radical resection	Dukes B2/C after radical resection	Cisplatin/MMC/CF/5-FU plus thymosin alpha 1, 1.6 mg IM daily on days 8–11 and 15–18	Same chemotherapy	NA	First 4-week cycle	⑥⑦⑬	Cellular and humoral immunity
Zhang H 2005 (RCT) (60)	26	26	NR; overall median 70.5 (60–75)	NR; overall median 70.5 (60–75)	Advanced colon cancer	Advanced colon cancer	5-FU/DDP/CF plus thymosin alpha 1, 1.6 mg daily for 5 days before chemotherapy, then three times weekly	Same chemotherapy	NA	2 cycles, 21 days each	⑧⑨⑩	Quality of life and toxicity
Chen 2001 (RCT) (61)	14	11	69.62 (65–80)	70.32 (66–79)	Rectal cancer; stage NR	Rectal cancer; stage NR	Dixon surgery plus thymalfasin, 1.6 mg SC daily for 4 days before surgery, then every other day until discharge	Same surgery, nutrition and antibiotics	NA	NA	⑪	Postoperative bowel dysfunction
Jiang 2009 (RCT) (62)	30	30	48.2 ± 9.2	45.2 ± 8.9	Metastatic colorectal cancer	Metastatic colorectal cancer	Oxaliplatin/LV/5-FU plus thymosin alpha 1, 1.6 mg SC every 3 days until chemotherapy completion	Same chemotherapy	NA	4 courses; 8 administrations every 2 weeks	①②⑥⑦⑬	Immune function and survival
Zhang X 2009 (RCT) (63)	40	36	55.4 (36–80)	55.6 (37–79)	After radical colon resection; stage NR	After radical colon resection; stage NR	FOLFOX4 plus thymopentin, 10 mg IV daily for 7 days per cycle	FOLFOX4; same G-CSF rescue rules	NA	6 cycles, every 2 weeks	⑥⑬	Immune function and blood counts
Zhang HH 2026 (RCT) (64)	33	33	63.48 ± 10.47	65.52 ± 8.95	Stage II/III: 15/18; after chemotherapy	Stage II/III: 18/15; after chemotherapy	Thymalfasin, 1.6 mg SC twice weekly for 8 weeks after standard adjuvant chemotherapy	Matched herbal placebo after standard adjuvant chemotherapy	NA	Completed; concurrent chemotherapy NA	⑥⑦⑧⑨⑩⑬	Immune recovery; safety and quality of life

RCT, randomized controlled trial; NRSI, nonrandomized study of an intervention; NR, not reported; NA, not applicable; T, thymosin group; C, control group. Outcome codes: ① OS; ② DFS/PFS; ③ fixed-time survival; ④ recurrence/metastasis; ⑤ ORR/DCR; ⑥ T-cell subsets; ⑦ NK cells; ⑧ KPS/quality of life; ⑨ hematologic toxicity; ⑩ nonhematologic toxicity; ⑪ infection/postoperative complications; ⑫ radiation enteritis; ⑬ exploratory biomarkers. Counts refer to eligible comparison arms. Overall values are identified when group-specific information was unavailable. The continuation provides clinical context and assessment windows.

**Table 2:** Clinical context, assessment windows and adjustment information.

Study	Male/female T; C	Center and recruitment	Site, stage and disease extent	Treatment intent	Assessment and follow-up	Adjustment
Ren 2024	T 20/36; C 15/35	Shanxi Provincial People's Hospital 2019 Apr–2023 Aug	Colon/rectum T 36/20, C 27/23; II/III: T 38/18; C 30/20	Postoperative adjuvant	After 6 XELOX cycles; reported 2-year follow-up, chronology unclear	NR
Yang 2025	T 35/16; C 32/19	Second Affiliated Hospital of Chengdu Medical College / Nuclear Industry 416 Hospital 2020 Jan–2024 Jan	Rectum; High-risk II after radical resection; some received neoadjuvant chemoradiotherapy	Postoperative adjuvant	Immune: 6 months; median follow-up 29 months	NA
Zhang 2023	T 66/54; C 64/56	Eastern campus of Ningbo Medical Center Lihuili Hospital 2018 Jun–2020 Jun	Colon/rectum: T 64/56; C 66/54; III/IV: T 72/48; C 74/46	Postoperative stage III/IV	6–8 XELOX cycles; reported 3-year follow-up, chronology unclear	NA
Huang 2019	T 10/15; C 11/14	Yingde People's Hospital 2016 Aug–2018 Jun	Rectum; II/IIIA/IIIB: T 4/7/14; C 5/7/13; no distant metastasis	First postoperative chemotherapy course	KPS: days 4/7 average; WBC: days 1/7	NA
Yang 2018	Overall 26/29	Shanghai East Hospital affiliated to Tongji University 2013 Jun–2014 Jun	Colorectum: overall colon/rectum 24/31; II/III: overall 25/30	Postoperative adjuvant	Treg/mRNA: 6/12 months; randomized 28/27, analyzed 26/23	NA
Liu 2018	NR	Yancheng Hospital of Traditional Chinese Medicine affiliated to Nanjing University of Chinese Medicine 2015 May–2017 May	Colorectum; IV	Advanced systemic treatment	After 4 XELOX cycles; KPS dispersion unclear	NA
Chen 2018	T 25/15; C 29/12	Huzhou First People's Hospital affiliated to Huzhou University 2009 May–2014 Jul	Colon/rectum: T 26/14; C 22/19; II/III: T 15/25; C 17/24	Postoperative adjuvant	Immune monthly; final KPS/immune at 6 months	NR
Xu 2016	Conflict: text 29/13 overall; **Table 1** totals 20/22	Chinese PLA General Hospital 2015 Jan–Apr	Rectum; I–III; abdominoperineal radical resection	Perioperative surgery; no chemoradiotherapy	Preoperative day 1; postoperative days 1/5; final day 5	NR
Zhou 2015	T 14/11; C 15/10; overall text total inconsistent	Mianyang Third People's Hospital 2009 Apr–2012 Aug	Colorectum; IV	Stage IV systemic treatment	After treatment; exact day NR; 8 XELOX cycles planned	NA
Liang 2015	NR	Baotou Cancer Hospital 2008 Oct–2010 May	Colon; II/III: T 6/14; C 5/15	Maintenance after adjuvant chemotherapy	Post-maintenance immune/KPS; 3-/5-year OS; denominators unclear	NA
Song 2013	T 22/18; C 23/17	Tancheng First People's Hospital 2006 Aug–2009 Jun	Colorectum; I–III/IV: T 23/17; C 22/18	Mixed stages I–IV; intent not separable	Immune: 2 weeks after chemotherapy; KPS: post-treatment; survival: 1/2/3 years	NA
Shi 2013	T 33/27; C 35/25	Recruitment center NR; affiliations in Zhuhai and Harbin 2010 Dec–2012 Apr	Colon; III after surgery	Postoperative adjuvant	Blood: 1 day after cycle 2; KPS improvement threshold ≥10 points	NA
Chai 2012	NR	First Hospital of Shanxi Medical University 2011 Sep–2012 Apr	Colorectum; III–IV	Advanced systemic treatment	Blood: 1–2 days before and 3 weeks after chemotherapy	NA
Dai 2012	Overall 35/29	Northern Jiangsu People's Hospital 2006 May–2009 May	Colon/rectum 33/31 overall; II/IIIA/IIIB 14/18/32 overall; no distant metastasis	First postoperative chemotherapy course	Immune after treatment, day NR; KPS days 1/3/5 average; WBC days 1/3/7 average	NA
Sheng 2011	Overall 44/30	First Affiliated Hospital of Xiamen University 2009 Jul–2010 Aug	Colon/rectum 47/27 overall; Radical resection 22; unresectable/incomplete resection 20; recurrent/metastatic 32	Mixed adjuvant/advanced treatment	Immune: 1 week after course 4	NA
Tao 2009	Overall 27/15	Renmin Hospital of Wuhan University and Wuhan Pukang Hospital 2000 Oct–2005 Dec	Colon/rectum 28/14 overall; IIA/IIB/IIIA/IIIB 13/16/7/6 overall after radical resection	Postoperative adjuvant and prolonged adjunct	Immune: 1/2/3 years; recurrence denominators unclear	NA
Long 2008	Overall 32/6	Naxi District People's Hospital, Luzhou NR	Rectum; III/IV 32/6 overall	Regional infusion; mixed advanced stages	After 3 courses; clinical analysis 26/10, immune denominator NR	NA
Huo 2011	Overall 46/30	Dalian Municipal Central Hospital 2007 Jan–2010 Aug	Colon/rectum 36/40 overall; IIB/IIIA/IIIB 20/34/22 overall	Postoperative adjuvant	After 2 cycles; exact immune sampling day NR	NA
Wu 2017	Overall 56/24	Dongying Second People's Hospital 2016 Jan–2017 Jan	Colon/rectum 40/40 overall; Advanced disease; precise TNM NR	Advanced systemic treatment	Cumulative toxicity during 2 XELOX courses; grading NR	NA
Yan 2023	T 27/12; C 24/17	Affiliated Hospital of Qingdao University 2020 Oct–2022 Sep	Rectum; postoperative pII/IIIA/IIIB/IIIC: T 6/2/27/4, C 10/2/26/3; positive margins T 4, C 2; no distant metastasis	Postoperative chemoradiotherapy	Immune: 1 week after radiotherapy; toxicity through that week	NA
Shi X 2018	T 20/10; C 18/12	Liyang Branch of Jiangsu Provincial People's Hospital 2015 Jun–2017 Jun	Colorectum; II/III/IV mixed; postoperative or resected primary advanced disease	Mixed-stage first-line chemotherapy	After 2 cycles; performance scale ZPS, not KPS	NA
Yao 2024	NR	Beijing Friendship Hospital 2021 Mar–2022 Dec	Low rectum; Locally advanced; precise TNM NR in abstract	Neoadjuvant chemoradiotherapy + PD-1 inhibitor	Postoperative pathological response; toxicity: CTCAE 4.0 during treatment	NR
Niu 2024	Combined T 124/76; C 119/81	Fourth Hospital of Hebei Medical University 2019 Feb–2021 Jan	Colorectum; cT1+ or N+ eligibility criterion; precise pathological stage and metastatic distribution NR	Perioperative and postoperative adjuvant	Immune day NR; complications: 30 days/12 months; 3-year follow-up incomplete	NA
Sha 2024	T 51/43; C 44/42	Shuguang Hospital affiliated to Shanghai University of Traditional Chinese Medicine 2017 Jan–2019 Dec	Colon/rectum: T 25/69; C 23/63; II/III: T 67/27; C 55/31	Postoperative adjuvant	After 4 cycles; recurrence at 1/3 years	NR
Xu 2004	T 21/8; C 14/6	Nanjing General Hospital of Nanjing Military Command 2000 Jan–2001 Jun	Colorectal; Dukes B2/C after radical resection	First postoperative chemotherapy course	Immune: days 8/22 of cycle 1; day 22 displayed	NA
Zhang H 2005	Overall 42/10	Henan Provincial People's Hospital NR	Colon; Advanced colon cancer	Advanced colon cancer systemic treatment	2 courses; QOL before/15 days after each course; WHO toxicity grades	NA
Chen 2001	T 5/9; C 4/7	First Affiliated Hospital of Wenzhou Medical College 1998 Jun–2001 Apr	Rectum; after Dixon resection; stage NR	Perioperative recovery after Dixon surgery	During admission; randomized 14/11; bowel-symptom analysis 14/9	NA
Jiang 2009	T 15/15; C 16/14	Zhejiang Xiaoshan Hospital 2005 Jan–2006 Dec	Colorectal; Metastatic colorectal cancer	Measurable metastatic disease	Immune: day 8 after course 4; clinical follow-up every 3 months for 2 years	NA
Zhang X 2009	NR	Jiamusi Central Hospital 2007 Jan–2008 Dec	Colon; after radical resection; stage NR	Adjuvant after radical colon resection	Immune before/after cycles; summarized cycle NR; weekly blood counts for 3 weeks	NA
Zhang H 2026	T 19/14; C 17/16	Three centers; coordinating First Affiliated Hospital of Anhui Medical University 2021 Jan–2024 Jun	Colorectal; Stage II/III: 15/18; after chemotherapy; C: Stage II/III: 18/15; after chemotherapy	Recovery after completed chemotherapy	8 weeks; eligible thymalfasin/placebo arms 33/33	NA

NR, not reported; NA, not applicable. Sex is reported as male/female, with group-specific or overall values as available. Conflicting source descriptions are identified without inferring missing values. Adjustment refers to variables in an adjusted between-group effect model; no usable adjusted estimate was available for the analyzed nonrandomized comparisons.

Among the 25 RCTs, 19 had some concerns and six had high overall risk of bias for the selected results. Limitations concerned allocation procedures, deviations from treatment, missing data, outcome reporting and source-reporting concerns. All five nonrandomized studies had serious overall risk of bias, principally from confounding. **Figure 2** presents the design-specific assessments.

**Figure 2:**
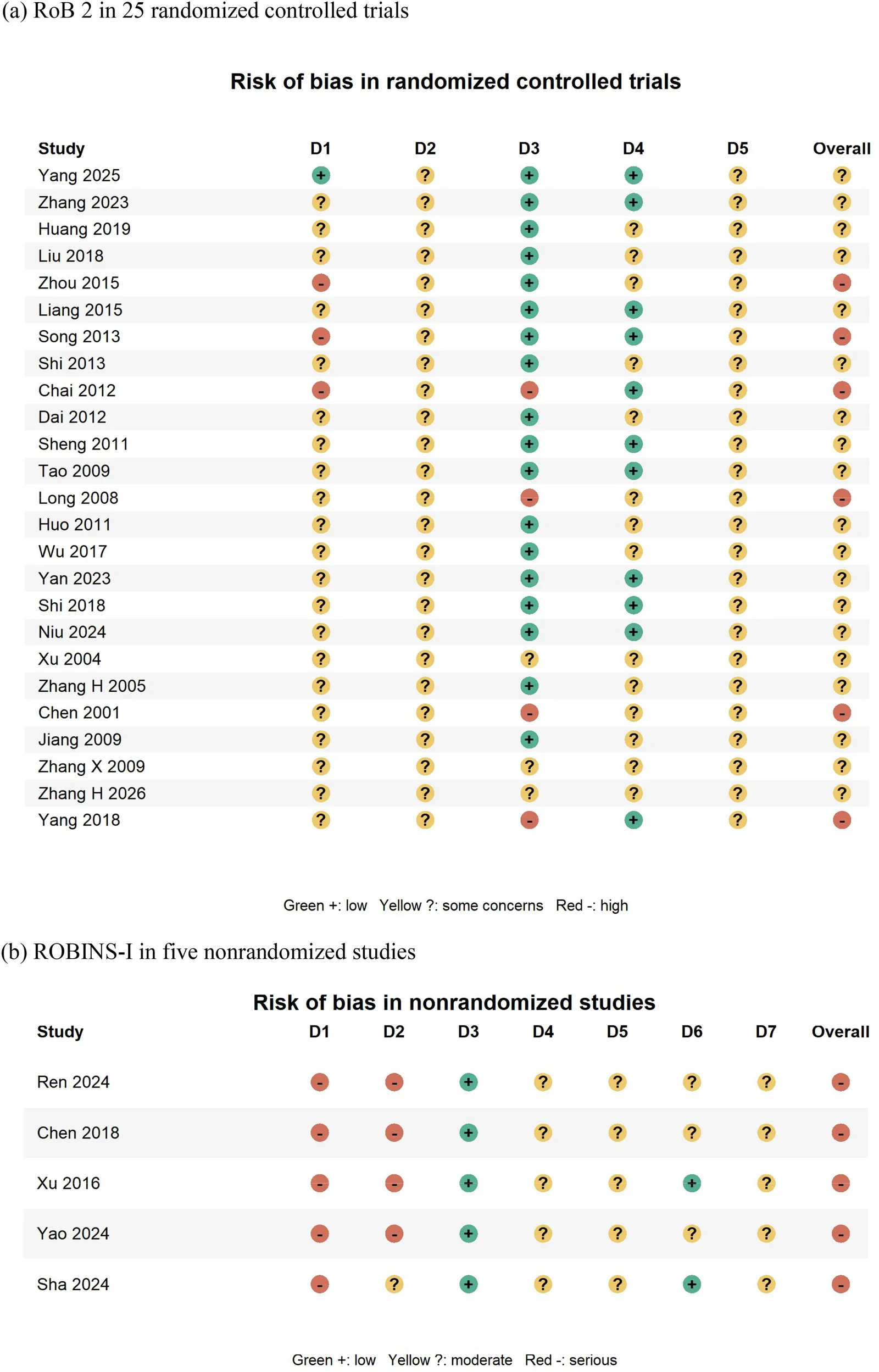
Risk-of-bias assessments. **(a)** RoB 2 in 25 randomized controlled trials. Assessments refer to the selected study outcomes. **(b)** ROBINS-I in five nonrandomized studies.

### 3.3 Postoperative treatment

In postoperative RCTs, CD4 percentage was higher with thymosin (six studies, 785 participants; MD 6.33 percentage points, 95% CI 4.89 to 7.76; I² = 58%), as was the CD4/CD8 ratio (seven studies, 887 participants; MD 0.25, 0.16 to 0.35; I² = 82%). CD3 (four studies, 589 participants; MD 3.26, −1.80 to 8.31; I² = 91%) and CD8 percentages (five studies, 709 participants; MD 1.52, −0.92 to 3.96; I² = 85%) were inconclusive. Separately, three nonrandomized studies (367 participants) showed higher CD4 percentage (MD 4.41, 0.93 to 7.88; I² = 96%) and CD4/CD8 ratio (MD 0.22, 0.04 to 0.40; I² = 96%) and lower CD8 percentage (MD −1.16, −2.00 to −0.31; I² = 50%). These included the six-month assessment in Chen 2018. One nonrandomized study reported higher CD3 percentage (180 participants; MD 3.79, 1.50 to 6.08). These unadjusted associations were not combined with the RCT estimates (**Figure 3**).

**Figure 3:**
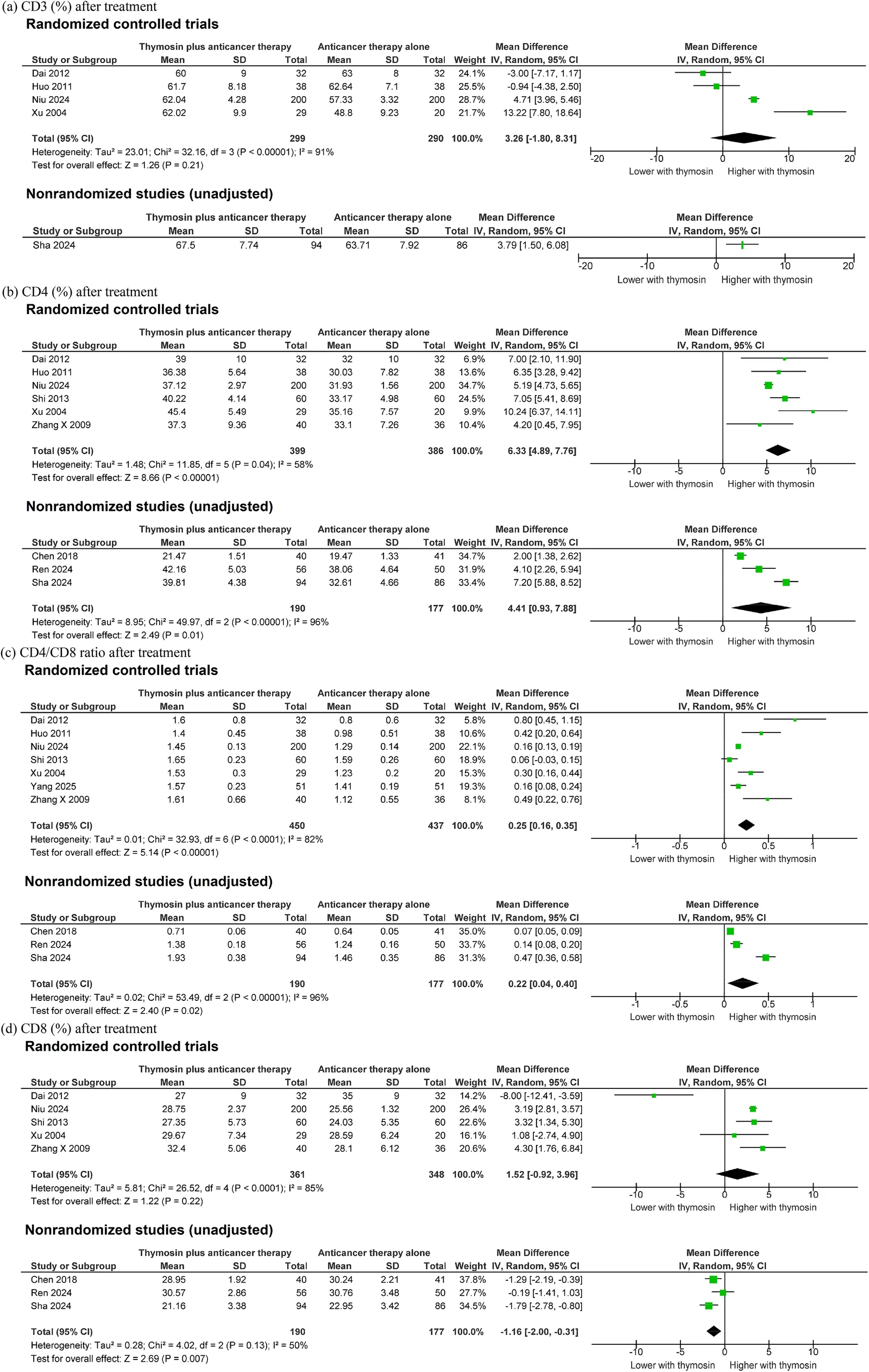
Postoperative T-cell indices. **(a)** CD3 (%) after treatment. **(b)** CD4 (%) after treatment. **(c)** CD4/CD8 ratio after treatment. **(d)** CD8 (%) after treatment. Mean differences are percentage points, except for the CD4/CD8 ratio.

Two RCTs reported higher early postoperative KPS (114 participants; MD 8.50 points, 95% CI 6.52 to 10.48; I² = 0%); two nonrandomized studies separately yielded MD 4.76 (187 participants; 2.58 to 6.94; I² = 0%). NK-cell percentage was higher in four RCTs (309 participants; MD 6.41 percentage points, 3.72 to 9.11; I² = 92%). An exploratory white-cell-count synthesis included three RCTs (190 participants; MD 1.25 × 10^9^/L, 0.02 to 2.49; I² = 94%), with different sampling schedules (**Figure 4**). For postoperative toxicity, one RCT reported inconclusive neutropenia (240 participants; RR 0.91, 0.82 to 1.01), lower liver injury (RR 0.50, 0.30 to 0.83) and lower thrombocytopenia (RR 0.75, 0.59 to 0.96). These were combined WHO grades I–IV. Two nonrandomized studies reported lower unadjusted neutropenia risk (286 participants; RR 0.65, 0.51 to 0.83), but inconclusive liver dysfunction (RR 0.52, 0.15 to 1.88). Nonrandomized thrombocytopenia evidence comprised one study (180 participants; RR 1.07, 0.67 to 1.72; **Figure 5**). Ren 2024 reported unspecified cytopenia (7/56 versus 5/50), which was not pooled with thrombocytopenia. In a separate perioperative study without concurrent chemotherapy, Xu 2016 reported postoperative-day-5 CD4 percentages of 26.84 ± 2.82 versus 24.55 ± 2.67 (21 participants per arm). Its acute surgical context was not combined with postoperative chemotherapy comparisons.

**Figure 4:**
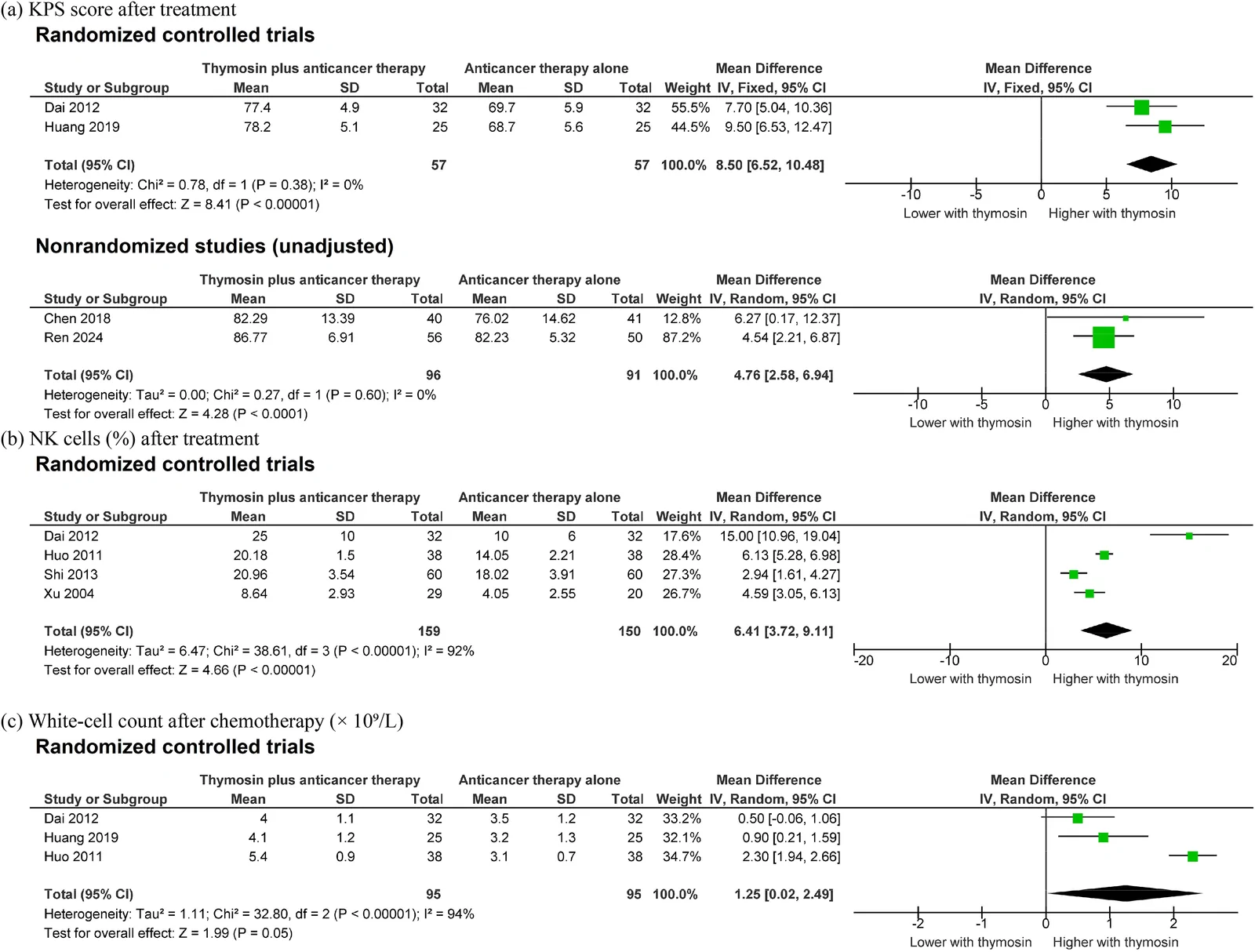
Postoperative functional status, NK-cell percentage, and white-cell count. **(a)** KPS score after treatment. **(b)** NK cells (%) after treatment. **(c)** White-cell count after chemotherapy (× 10^9^/L). KPS, Karnofsky Performance Status; NK, natural killer; WBC, white blood cells.

**Figure 5:**
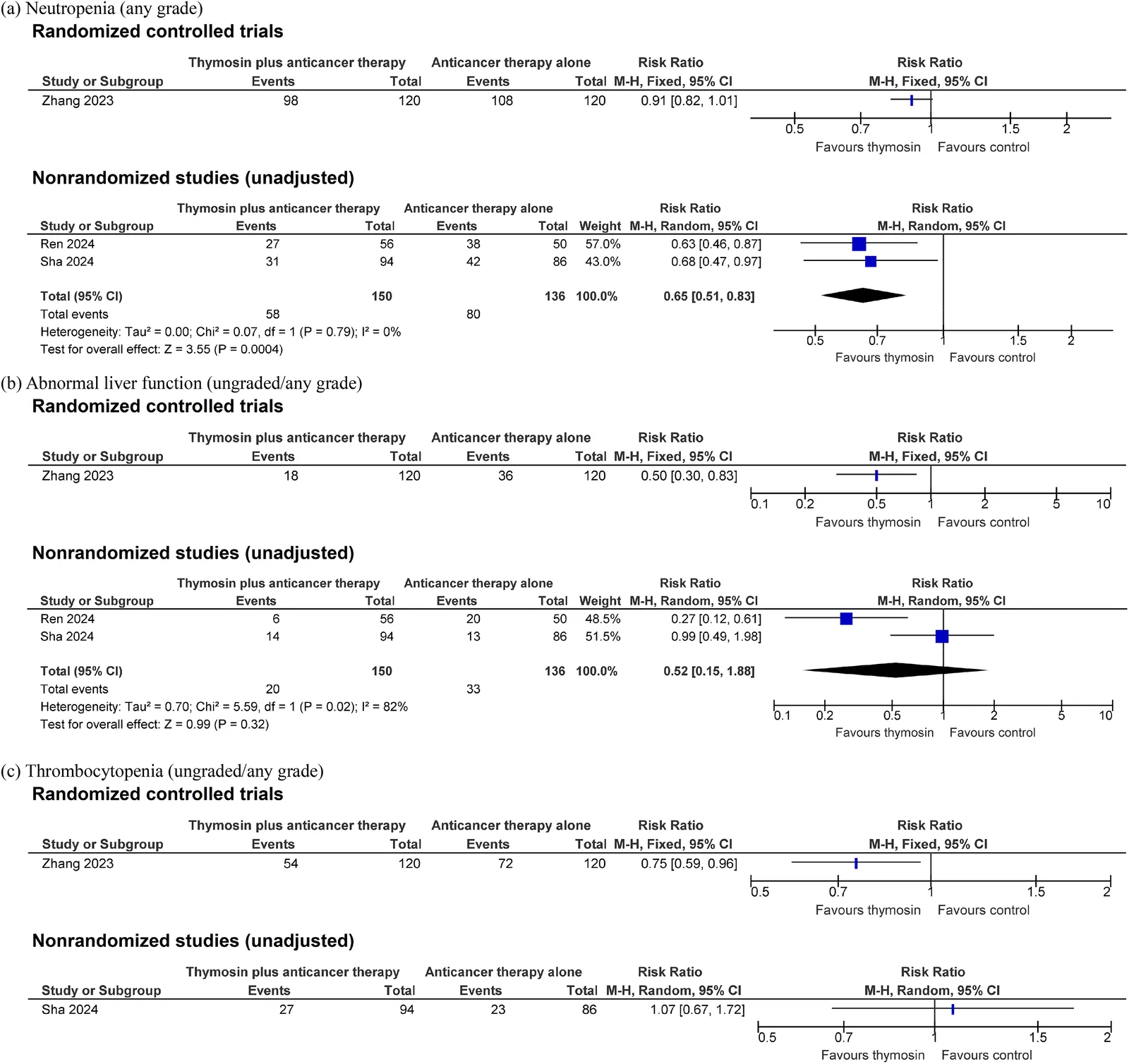
Postoperative toxicity outcomes. **(a)** Neutropenia (any grade). **(b)** Abnormal liver function (ungraded/any grade). **(c)** Thrombocytopenia (ungraded/any grade). RCT events are WHO grades I–IV; nonrandomized events are ungraded.

Additional postoperative comparisons addressed distinct clinical periods. Yan 2023 assessed patients one week after radiotherapy: KPS was 81.02 ± 5.98 versus 78.29 ± 5.432 (39/41 participants), and grade 3 acute radiation enteritis occurred in 0/39 versus 3/41. These radiation-specific findings were not pooled with chemotherapy-period outcomes. Tao 2009 reported CD4 means of 40.45 versus 25.78 at three years, a substantially later window. Yang 2025 reported recurrence/metastasis in 7/51 versus 16/51 over a median 29-month follow-up; variable follow-up and losses limited interpretation of these crude proportions. Longer-term survival summaries without compatible effect estimates and immune results with unresolved group labels or denominators were not pooled.

### 3.4 Advanced, metastatic, or mixed-stage disease

In advanced, metastatic or mixed-stage disease, Song 2013 supplied the only eligible one-year survival comparison (80 participants; RR 1.52, 95% CI 1.18 to 1.95) (45). Three RCTs contributed CD3 (214 participants; MD 9.95 percentage points, 5.95 to 13.96; I² = 76%) and CD4 percentages (MD 9.19, 7.99 to 10.39; I² = 0%). CD8 percentage was inconclusive (MD −4.04, −10.69 to 2.61; I² = 97%). Two trials contributed the CD4/CD8 ratio (154 participants; MD 0.49, 0.42 to 0.57; I² = 2%; **Figure 6**). These analyses include mixed-stage cohorts in Song 2013 and Sheng 2011 and therefore do not represent exclusively advanced disease. Two thymopentin trials reported higher KPS (130 participants; MD 4.35 points, 1.90 to 6.80; I² = 0%). Both original reports give SDs of 7.52 and 6.69 in the intervention and control arms; the concordance is present in the publications. Their differing sample sizes, stage distributions and treatment periods warrant caution. NK-cell percentage was inconclusive (three trials, 214 participants; MD 9.16, −2.18 to 20.50; I² >99%; **Figure 7**). Liu 2018 reported KPS means of 82.15 and 76.53, but its dispersion definition was unclear; a CI was not calculated from an assumed SD.

**Figure 6:**
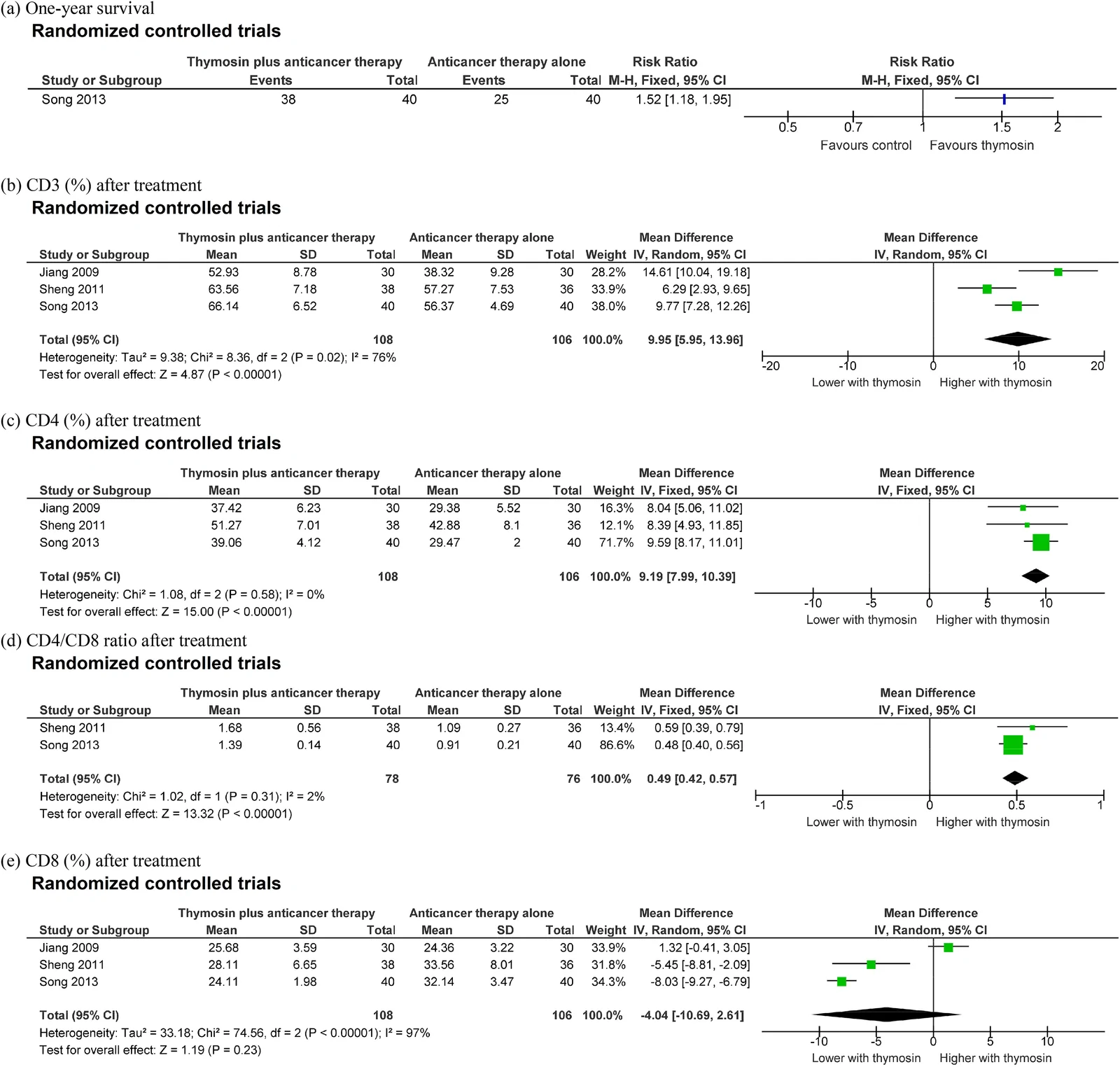
One-year survival and T-cell indices in advanced or mixed-stage disease. **(a)** One-year survival. **(b)** CD3 (%) after treatment. **(c)** CD4 (%) after treatment. **(d)** CD4/CD8 ratio after treatment. **(e)** CD8 (%) after treatment. Survival is a single-study estimate.

**Figure 7:**
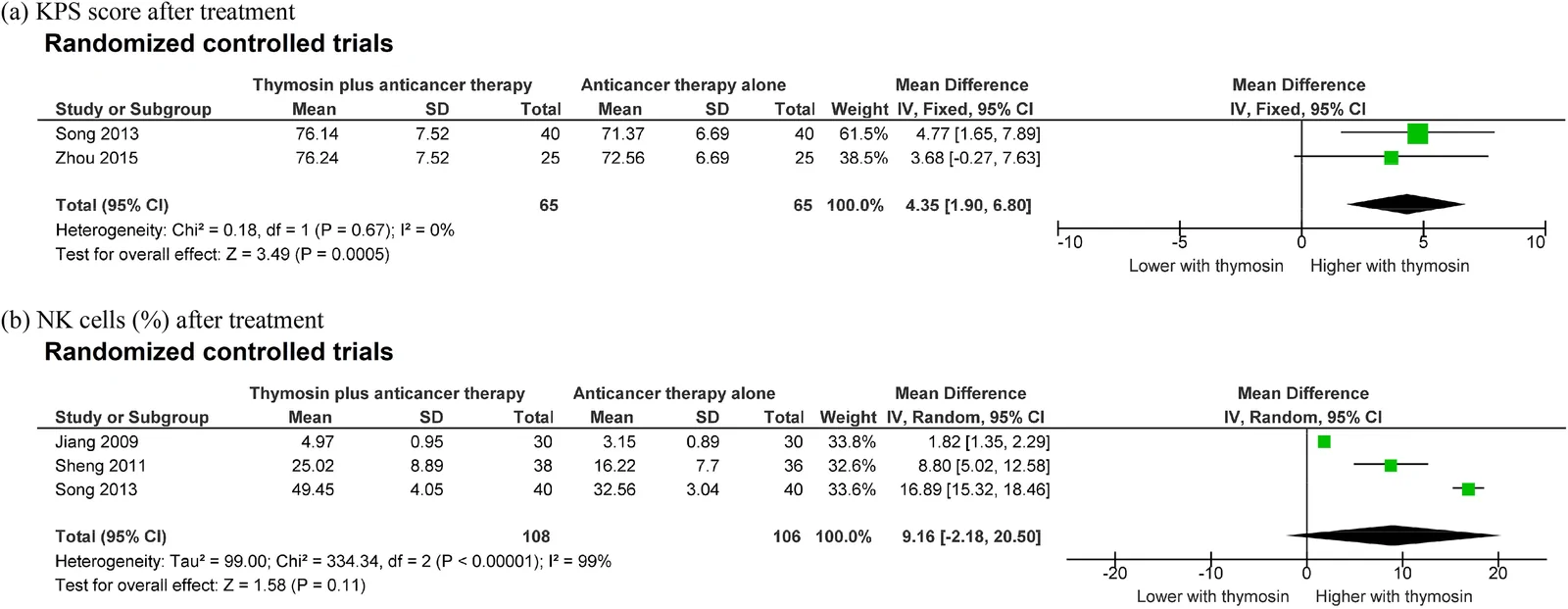
KPS and NK-cell percentage in advanced or mixed-stage disease. **(a)** KPS score after treatment. **(b)** NK cells (%) after treatment. KPS, Karnofsky Performance Status; NK, natural killer.

Hand–foot syndrome was less frequent in two thymopentin-plus-XELOX RCTs (130 participants; RR 0.37, 95% CI 0.17 to 0.82). Neurotoxicity (RR 0.94, 0.69 to 1.30), granulocytopenia (RR 0.79, 0.61 to 1.02) and gastrointestinal reactions (RR 0.90, 0.66 to 1.21) were inconclusive. Three RCTs contributed liver dysfunction (182 participants; RR 0.81, 0.66 to 1.01) and thrombocytopenia (RR 0.62, 0.28 to 1.41); these analyses also included thymosin alpha 1 with 5-fluorouracil/cisplatin/leucovorin and do not represent thymopentin alone (**Figure 8**). The toxicity outcomes overlap within the same participants. Any-grade and ungraded events cannot establish a reduction in severe or drug-attributable events. Yao 2024, a nonrandomized neoadjuvant chemoradiotherapy-plus-PD-1 study, reported pathological complete response in 8/13 versus 5/13 participants and any adverse event in 11/13 versus 10/13. This small, unadjusted comparison was considered separately from chemotherapy-only settings.

**Figure 8:**
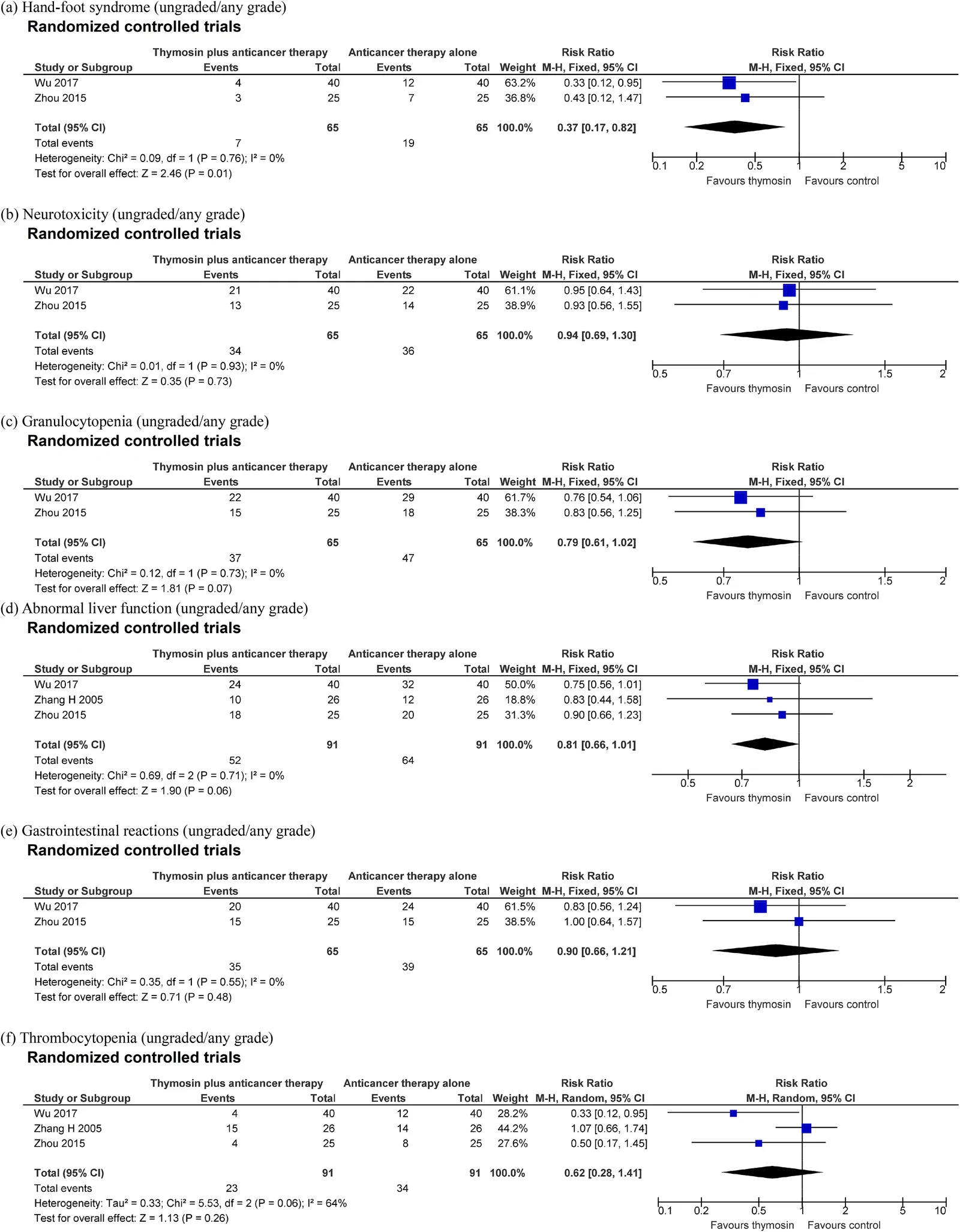
Toxicity outcomes in advanced or mixed-stage disease. **(a)** Hand-foot syndrome (ungraded/any grade). **(b)** Neurotoxicity (ungraded/any grade). **(c)** Granulocytopenia (ungraded/any grade). **(d)** Abnormal liver function (ungraded/any grade). **(e)** Gastrointestinal reactions (ungraded/any grade). **(f)** Thrombocytopenia (ungraded/any grade). Events are any-grade or ungraded, as reported.

Shi X 2018 reported higher post-treatment CD4 (45.73 versus 36.45) and CD4/CD8 (1.62 versus 1.05) and lower CD8 (30.04 versus 35.23) after two chemotherapy cycles in 30 participants per arm. Inconsistencies between baseline tables and figures and repeated numerical values limited quantitative interpretation, and these data were described separately. Its performance-status scale was ZPS rather than KPS.

### 3.5 Sensitivity analysis

Twelve RCT outcomes underwent leave-one-out analysis. Postoperative CD4, CD4/CD8 ratio and NK-cell estimates remained positive after each omission. CD3 and CD8 estimates were less stable, and the white-cell-count result was sensitive to omission. Advanced-disease thrombocytopenia was also sensitive to individual studies (**Figure 9**). Excluding studies with unclear assessment timing retained positive postoperative CD4 (MD 7.28, 95% CI 5.98 to 8.59) and CD4/CD8 findings (MD 0.29, 0.13 to 0.45). Restricting white-cell counts to the two early-window studies gave MD 0.66 × 10^9^/L (0.22 to 1.10). For KPS, omission of Song 2013 left Zhou 2015 alone (MD 3.68, −0.27 to 7.63); omission of Zhou 2015 left Song 2013 alone (MD 4.77, 1.65 to 7.89). Excluding both left no poolable thymopentin KPS data. After excluding Song, two-trial estimates remained positive for CD3 (MD 10.30, 95% CI 2.15 to 18.45) and CD4 (MD 8.19, 5.93 to 10.45), while CD8 and NK remained inconclusive; CD4/CD8 was then a single-study result from Sheng 2011. The three nonrandomized immune syntheses were sensitive to individual-study omission: CIs included no difference in at least one omission analysis for each of CD4, CD4/CD8 and CD8. These analyses limit reliance on individual studies and apparently repeated signals from the affected reports.

**Figure 9:**
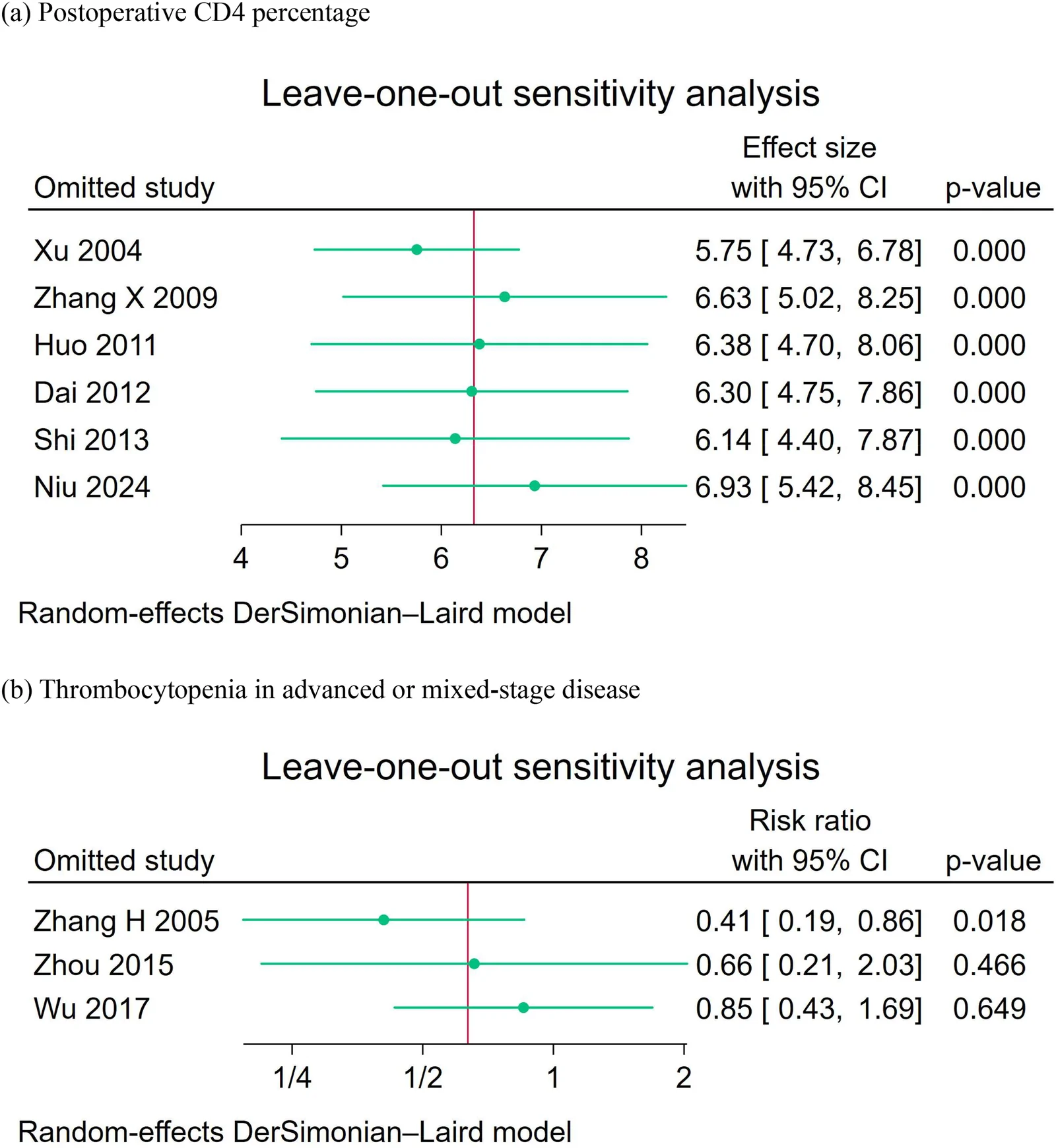
Leave-one-out sensitivity analyses. **(a)** Postoperative CD4 percentage. **(b)** Thrombocytopenia in advanced or mixed-stage disease. RCTs only; random-effects RRs use inverse-variance estimation in Stata.

### 3.6 Certainty of evidence

RCT certainty was low for postoperative CD4, postoperative KPS and toxicity outcomes, and advanced-disease hand–foot syndrome, neurotoxicity, granulocytopenia, liver dysfunction and gastrointestinal reactions. Postoperative CD4 was downgraded for risk of bias and inconsistency; the other low-certainty outcomes were downgraded for risk of bias and sparse information or imprecision. The remaining RCT estimates were very low certainty. Marked heterogeneity and limited information added to bias concerns for the remaining postoperative markers, white-cell counts and advanced-disease thrombocytopenia. Source-reporting concerns warranted two-level risk-of-bias downgrading for Song-related survival and immune estimates and Song/Zhou KPS, with further downgrading for imprecision or inconsistency as applicable. All nonrandomized estimates were very low certainty because of serious residual confounding, with additional heterogeneity or imprecision where present. **Table 3** reports certainty separately for each design and outcome; concordance between designs was not used to upgrade certainty.

**Table 3:** Effect estimates and certainty of evidence by treatment setting and study design.

Setting / design	Outcome	k / n	Effect (95% CI)	I²	Model	Certainty
Postoperative RCT	CD3 (%) after treatment	4 / 589	MD 3.26 (-1.80 to 8.31)	91%	Random	Very low
Postoperative NRSI	CD3 (%) after treatment	1 / 180	MD 3.79 (1.50 to 6.08)	NA	Single	Very low
Postoperative RCT	CD4 (%) after treatment	6 / 785	MD 6.33 (4.89 to 7.76)	58%	Random	Low
Postoperative NRSI	CD4 (%) after treatment	3 / 367	MD 4.41 (0.93 to 7.88)	96%	Random	Very low
Postoperative RCT	CD4/CD8 ratio after treatment	7 / 887	MD 0.25 (0.16 to 0.35)	82%	Random	Very low
Postoperative NRSI	CD4/CD8 ratio after treatment	3 / 367	MD 0.22 (0.04 to 0.40)	96%	Random	Very low
Postoperative RCT	CD8 (%) after treatment	5 / 709	MD 1.52 (-0.92 to 3.96)	85%	Random	Very low
Postoperative NRSI	CD8 (%) after treatment	3 / 367	MD -1.16 (-2.00 to -0.31)	50%	Random	Very low
Postoperative RCT	KPS score after treatment	2 / 114	MD 8.50 (6.52 to 10.48)	0%	Fixed	Low
Postoperative NRSI	KPS score after treatment	2 / 187	MD 4.76 (2.58 to 6.94)	0%	Random	Very low
Postoperative RCT	NK cells (%) after treatment	4 / 309	MD 6.41 (3.72 to 9.11)	92%	Random	Very low
Postoperative RCT	Neutropenia (any grade)	1 / 240	RR 0.91 (0.82 to 1.01)	NA	Single	Low
Postoperative NRSI	Neutropenia (any grade)	2 / 286	RR 0.65 (0.51 to 0.83)	0%	Random	Very low
Postoperative RCT	Abnormal liver function (ungraded/any grade)	1 / 240	RR 0.50 (0.30 to 0.83)	NA	Single	Low
Postoperative NRSI	Abnormal liver function (ungraded/any grade)	2 / 286	RR 0.52 (0.15 to 1.88)	82%	Random	Very low
Postoperative RCT	Thrombocytopenia (ungraded/any grade)	1 / 240	RR 0.75 (0.59 to 0.96)	NA	Single	Low
Postoperative NRSI	Thrombocytopenia (ungraded/any grade)	1 / 180	RR 1.07 (0.67 to 1.72)	NA	Single	Very low
Advanced / mixed RCT	One-year survival	1 / 80	RR 1.52 (1.18 to 1.95)	NA	Single	Very low
Advanced / mixed RCT	CD3 (%) after treatment	3 / 214	MD 9.95 (5.95 to 13.96)	76%	Random	Very low
Advanced / mixed RCT	CD4 (%) after treatment	3 / 214	MD 9.19 (7.99 to 10.39)	0%	Fixed	Very low
Advanced / mixed RCT	CD4/CD8 ratio after treatment	2 / 154	MD 0.49 (0.42 to 0.57)	2%	Fixed	Very low
Advanced / mixed RCT	CD8 (%) after treatment	3 / 214	MD -4.04 (-10.69 to 2.61)	97%	Random	Very low
Advanced / mixed RCT	KPS score after treatment	2 / 130	MD 4.35 (1.90 to 6.80)	0%	Fixed	Very low
Advanced / mixed RCT	NK cells (%) after treatment	3 / 214	MD 9.16 (-2.18 to 20.50)	99%	Random	Very low
Advanced / mixed RCT	Hand-foot syndrome (ungraded/any grade)	2 / 130	RR 0.37 (0.17 to 0.82)	0%	Fixed	Low
Advanced / mixed RCT	Neurotoxicity (ungraded/any grade)	2 / 130	RR 0.94 (0.69 to 1.30)	0%	Fixed	Low
Advanced / mixed RCT	Granulocytopenia (ungraded/any grade)	2 / 130	RR 0.79 (0.61 to 1.02)	0%	Fixed	Low
Advanced / mixed RCT	Abnormal liver function (ungraded/any grade)	3 / 182	RR 0.81 (0.66 to 1.01)	0%	Fixed	Low
Advanced / mixed RCT	Gastrointestinal reactions (ungraded/any grade)	2 / 130	RR 0.90 (0.66 to 1.21)	0%	Fixed	Low
Advanced / mixed RCT	Thrombocytopenia (ungraded/any grade)	3 / 182	RR 0.62 (0.28 to 1.41)	64%	Random	Very low
Postoperative RCT	WBC count after chemotherapy (10^9/L)	3 / 190	MD 1.25 (0.02 to 2.49)	94%	Random	Very low
Postoperative NRSI	Cytopenia, type unspecified	1 / 106	RR 1.25 (0.42 to 3.69)	NA	Single	Very low

RCT estimates are the primary evidence; nonrandomized estimates are unadjusted supplementary associations. No cross-design pooled effect is reported. k, contributing studies; n, participants in that comparison; I² is not applicable to a single study. Reasons for downgrading are described in Section 3.6.

### 3.7 Trial sequential analysis

For liver dysfunction in advanced or mixed-stage disease, 182 RCT participants represented 49.3% of the RIS of 369 under the 20% relative risk reduction assumption. For thrombocytopenia, the same accrued sample represented 3.6% of the RIS of 5,034. Neither analysis crossed an efficacy monitoring boundary; observed-effect analyses likewise did not cross a boundary (**Figure 10**). Postoperative neutropenia was not eligible for TSA because only one RCT contributed.

**Figure 10:**
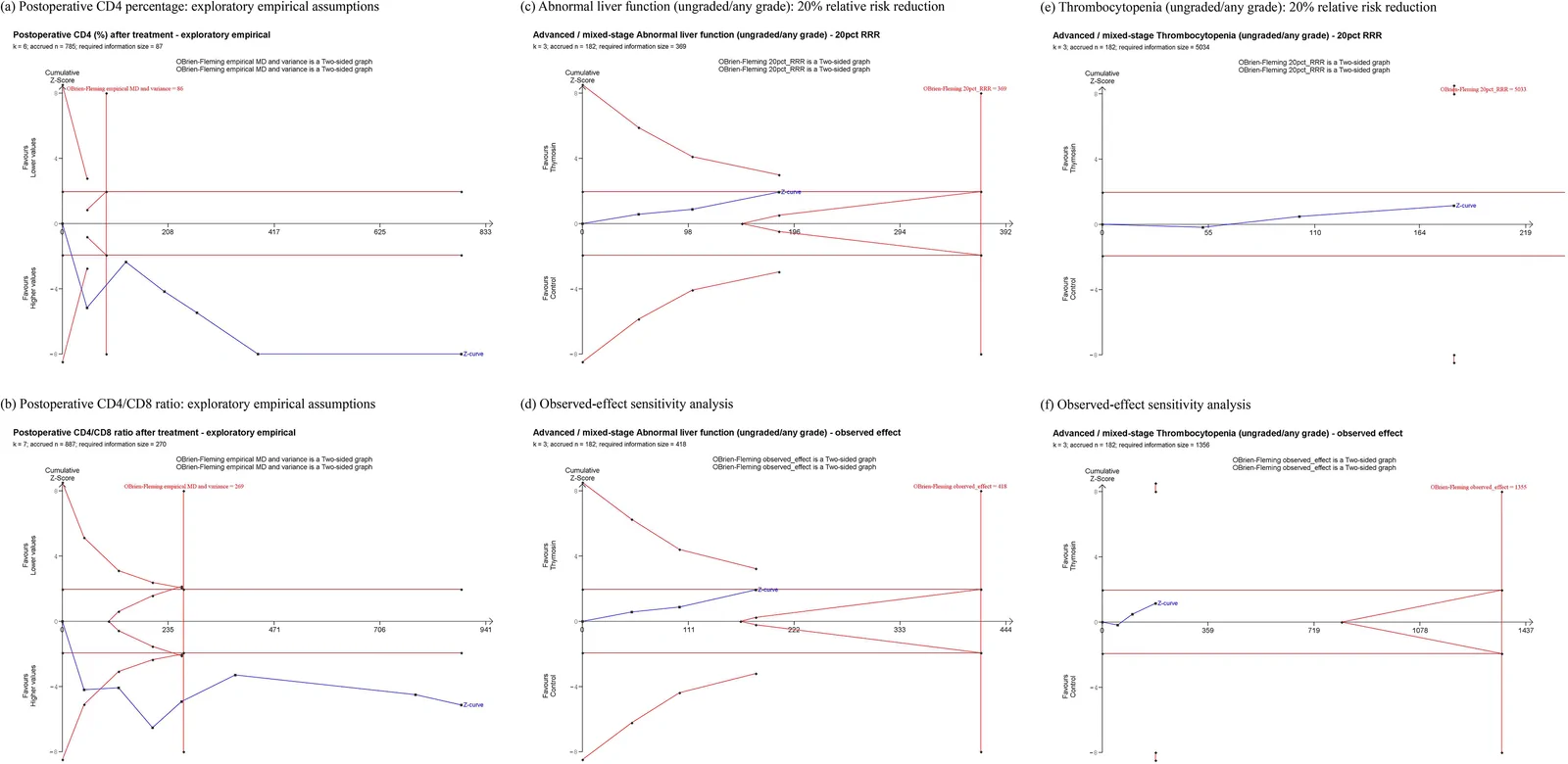
Trial sequential analyses. **(a)** Postoperative CD4 percentage: exploratory empirical assumptions. **(b)** Postoperative CD4/CD8 ratio: exploratory empirical assumptions. RCTs only; RIS, required information size; RRR, relative risk reduction. **(c)** Abnormal liver function (ungraded/any grade): 20% relative risk reduction. **(d)** Observed-effect sensitivity analysis. **(e)** Thrombocytopenia (ungraded/any grade): 20% relative risk reduction. **(f)** Observed-effect sensitivity analysis.

Exploratory continuous-outcome TSAs crossed an efficacy boundary for postoperative CD4, CD4/CD8 ratio and NK-cell percentage, whereas CD3 and CD8 remained below their information targets without boundary crossing. The empirical RIS estimates for CD4, CD4/CD8 and NK cells were approximately 87, 270 and 124 participants, respectively. These small, effect-dependent targets do not establish clinical benefit or overcome heterogeneity and risk of bias. Survival and KPS were not eligible for TSA. In advanced or mixed-stage disease, empirical TSA crossed a boundary for CD3 (RIS approximately 72), but not for CD8 or NK. For CD4, the empirical RIS of 13 participants was already exceeded by the first trial, so a sequential monitoring boundary was not estimable and no boundary-crossing claim was made. These highly effect-dependent analyses remain exploratory.

### 3.8 Publication bias and meta-regression

Each pooled outcome included fewer than ten studies. Publication bias and meta-regression could therefore not be evaluated reliably using the prespecified statistical methods.

## 4 Discussion

### 4.1 Principal findings and evidence pattern

This review brings together 30 controlled studies and distinguishes the evidence from 25 RCTs from five nonrandomized studies. RCT results suggest higher postoperative CD4 percentage, CD4/CD8 ratio and NK-cell percentage, while CD3 and CD8 findings remain uncertain. The persistence of several immune signals in sensitivity analyses provides a basis for more focused investigation. By separating study designs, the review also clarifies that the lower postoperative neutropenia risk observed in nonrandomized studies is not established by the available randomized comparison. This distinction is useful for identifying priorities for confirmatory trials.

KPS findings provide a possible signal of improved performance status, but their clinical importance and durability are uncertain. Statistical significance does not by itself establish a benefit that patients perceive as important. A pooled KPS mean difference describes a group-level contrast rather than the magnitude or frequency of meaningful individual improvement, and does not establish durability. Circulating immune-marker changes likewise cannot establish clinical benefit without validated links to patient-important outcomes. The early postoperative result comes from two small RCTs using different preparations, and the advanced or mixed-stage synthesis depends on two reports with identical SDs and different treatment periods. The hand–foot syndrome finding also warrants further evaluation, whereas most pooled advanced-disease toxicity estimates remain inconclusive. One postoperative RCT reported lower any-grade liver injury and thrombocytopenia, but these isolated findings do not establish a general safety advantage. Survival evidence is too limited to support a firm conclusion. The contribution of the review is therefore to define testable clinical signals and their evidential limits.

#### 4.2 Biological plausibility and endpoint hierarchy

Experimental findings provide a biological rationale for studying thymosin alpha 1. Alpha 1 can influence dendritic-cell maturation, antigen presentation, Toll-like receptor/MyD88 pathways and T-cell responses (20–22). Effects on macrophage polarization have also been reported (23). These mechanisms primarily concern alpha 1 and cannot automatically be extrapolated to thymopentin or heterogeneous thymic peptide injections. The clinical studies did not establish which mechanism accounted for the observed circulating changes.

Circulating CD3, CD4, CD8 and NK-cell percentages and the CD4/CD8 ratio are correlated, compositional measures and exploratory peripheral-blood indices (65). Changes can reflect redistribution or denominator shifts without restoration of absolute cell numbers, functional immune activity or antitumor efficacy. Intratumoral T-cell density and location are distinct from circulating composition (12, 13). Multiple positive marker findings in the same participants therefore do not provide independent confirmation of immune competence or survival benefit.

The observed immune and performance-status signals justify testing whether a defined preparation improves treatment tolerance. Future trials should link circulating markers with absolute cell counts, functional assays, infection, hospitalization, delivered chemotherapy dose intensity, delays, severe adverse events and patient-reported outcomes. This would determine whether laboratory changes correspond to benefits that matter to patients.

#### 4.3 Clinical positioning and sources of heterogeneity

The review addresses a broad question concerning thymosin preparations added to matched background treatment. Maintaining separate postoperative and advanced/metastatic/mixed-stage categories provides an overview of this evidence, but the categories do not establish equivalence among preparations or clinical situations. Alpha-1 evidence predominates in postoperative immune analyses; thymopentin contributes much of the advanced or mixed-stage clinical evidence, while some toxicity analyses also contain alpha 1. Mixed-stage cohorts were classified explicitly when stage-specific results were inseparable. Differences in treatment intent, chemotherapy regimen, dose, duration and assessment windows remain important sources of heterogeneity. The small numbers within clinically coherent strata preclude reliable effect-modification analyses. The summary estimates should therefore be interpreted as exploratory averages across the contributing studies, not as interchangeable effects of all preparations.

For KPS, the postoperative RCTs used the same instrument during the first chemotherapy course, but Dai 2012 averaged days 1, 3 and 5, whereas Huang 2019 averaged days 4 and 7. The advanced or mixed-stage reports assessed KPS after treatment; Song 2013 included stages I–IV and four XELOX courses, whereas Zhou 2015 included stage IV disease and planned eight cycles, with exact assessment days incompletely specified. The nonrandomized postoperative studies assessed KPS at six months or after six cycles and were analyzed separately. Thus, these summaries describe performance status within broad treatment periods, rather than effects at a harmonized time point. Common use of KPS and low statistical heterogeneity do not establish comparable measurement procedures or clinical settings. The pooled mean differences remain exploratory and should not be interpreted as established clinically important or durable gains in functioning.

Separating study designs prevents serious-risk nonrandomized evidence from determining the primary randomized estimates. Nevertheless, incomplete allocation reporting, missing outcomes and source-reporting concerns remain within the RCT evidence. The nonrandomized studies lacked usable adjusted comparisons for the analyzed outcomes and remained vulnerable to confounding by stage, performance status, comorbidity and treatment selection. Agreement in effect direction across designs does not remove these biases or establish causality.

Toxicity findings require formulation-, regimen- and observation-period-specific interpretation. Several outcomes were measured in the same participants, and no prespecified primary safety endpoint supported treating them as independent confirmations. Planned background regimens were matched within comparisons, but delivered relative dose intensity, reductions, delays, supportive care, hospitalization and drug attribution were incompletely reported. Lower actual chemotherapy exposure could explain both fewer toxicities and higher KPS. TSA offers an additional description of accumulated information but cannot correct these limitations. Its empirical immune-marker targets and boundary crossings should not supersede the low or very low GRADE certainty.

### 4.4 Strengths, limitations, and priorities for future trials

The review integrates evidence from English- and Chinese-language sources that would otherwise be difficult to assess together. Its separation of randomized and nonrandomized results, consideration of eligible multiarm comparisons, and combined use of risk-of-bias assessment, sensitivity analyses and GRADE provide a clearer account of the evidence supporting each outcome. The clinical detail in **Table 1** helps identify which patient groups, preparations and assessment periods warrant targeted prospective study.

Most studies were small, incompletely reported and conducted in Chinese clinical settings, which limits generalizability. Formulation and assessment differences contribute to uncertainty beyond statistical heterogeneity. Similar numerical findings in primary reports, including the identical KPS SDs in Song 2013 and Zhou 2015, cannot be explained by accurate extraction alone; full independence remains uncertain. Some outcomes lacked clear dispersion measures, denominators or scale definitions and could not be pooled. Information on severe toxicity and actual treatment exposure was limited. Small numbers within outcomes and clinically coherent strata also restricted investigation of publication bias and effect modification. These limitations reduce confidence in the magnitude and clinical applicability of the observed signals. These reporting constraints limited comparable outcome extraction and assessment of alternative explanations, despite source verification and sensitivity analyses.

Future multicenter RCTs should specify the thymosin preparation and treatment setting, use concealed allocation, prospectively register clinically important outcomes and report all randomized participants. Survival, recurrence, severe toxicity, treatment completion and patient-reported quality of life should take priority, with immune indices as supporting exploratory outcomes. Trials should use standardized KPS assessment times and blinded outcome assessors where feasible, and justify prespecified clinically important target differences for the population and endpoint. Sample sizes should be based on a patient-important primary outcome rather than an empirical immune-marker effect. Recording actual chemotherapy exposure, treatment modifications and supportive care would help distinguish adjunctive benefit from differences in anticancer treatment received.

Nutritional and metabolic context also warrants attention. Ponnusamy et al. discuss links among high-fat diet, inflammation and colorectal carcinogenesis (66). This background supports documenting baseline nutritional and metabolic characteristics in future trials; it does not establish that these factors modify responses to thymosin.

## 5 Conclusion

This review identifies potential improvements in selected circulating immune indices and performance status and clarifies the distinction between randomized evidence and nonrandomized associations. These findings provide a useful basis for selecting defined preparations, treatment settings and patient-important outcomes for future trials. The low or very low certainty, source-reporting concerns and limited clinical outcome data mean that current evidence is insufficient to support routine clinical use of thymosin preparations in colorectal cancer.

## Data Availability

The original contributions presented in the study are included in the article/supplementary material. Further inquiries can be directed to the corresponding authors.
